# Exhaustion of the bone marrow progenitor cell reserve is associated with major events in severe limb ischemia

**DOI:** 10.1007/s10456-019-09666-0

**Published:** 2019-03-30

**Authors:** Hendrik Gremmels, Femke C. C. van Rhijn-Brouwer, Diana A. Papazova, Joost O. Fledderus, Martin Teraa, Marianne C. Verhaar, the JUVENTAS study group

**Affiliations:** 1 Department of Nephrology and Hypertension, University Medical Center Utrecht, Postal Box 85500, 3508 GA Utrecht, The Netherlands; 2 Department of Surgery, University Medical Center Utrecht, Utrecht, The Netherlands; 3 Department of Vascular Surgery, University Medical Center Utrecht, Utrecht, The Netherlands

**Keywords:** Cell therapy, Stem cells, Vascular biology, Risk factors, Peripheral vascular disease

## Abstract

Lower numbers of progenitor cells (PCs) in peripheral blood (PB) have been associated with cardiovascular events in high-risk populations. Therapies aiming to increase the numbers of PCs in circulation have been developed, but clinical trials did not result in better outcomes. It is currently unknown what causes the reduction in PB PC numbers: whether it is primary depletion of the progenitor cell reserve, or a reduced mobilization of PCs from the bone marrow (BM). In this study, we examine if PB and BM PC numbers predict Amputation-Free Survival (AFS) in patients with Severe Limb Ischemia (SLI). We obtained PB and BM from 160 patients enrolled in a clinical trial investigating BM cell therapy for SLI. Samples were incubated with antibodies against CD34, KDR, CD133, CD184, CD14, CD105, CD140b, and CD31; PC populations were enumerated by flow cytometry. Higher PB CD34^+^ and CD133^+^ PC numbers were related to AFS (Both Hazard Ratio [HR_event_] = 0.56, *p* = 0.003 and *p* = 0.0007, respectively). AFS was not associated with the other cell populations in PB. BM PC numbers correlated with PB PC numbers and showed similar HRs for AFS. A further subdivision based on relative BM and PB PC numbers showed that BM PC numbers, rather than mobilization, associated with AFS. Both PB and BM PC numbers are associated with AFS independently from traditional risk factor and show very similar risk profiles. Our data suggest that depletion of the progenitor cell reserve, rather than decreased PC mobilization, underlies the association between PB PC numbers and cardiovascular risk.

**Electronic supplementary material:**

The online version of this article (10.1007/s10456-019-09666-0) contains supplementary material, which is available to authorized users.

## Introduction

Patients with peripheral artery disease (PAD) show faster functional decline and higher rates of cardiovascular events than the general population [1]. The most advanced stage of PAD, Severe or Chronic Limb-Threatening Ischemia (SLI or CLTI), occurs when atherosclerotic occlusion of the arteries of the lower limb reaches a point where blood supply cannot meet metabolic demands of the tissue even in rest [2]. In addition to risk factors associated with vascular damage, the deterioration of endogenous vascular repair mechanisms contributes to progression of PAD and increased cardiovascular risk [3].

Progenitor Cells (PCs) in the circulation may represent such an endogenous repair mechanism; circulating PCs were shown to contribute to vascular repair in animal models of ischemia [4, 5]. PB PCs are commonly identified by the progenitor cell markers CD34 and CD133. In addition, PCs are often stained for the presence of KDR (vascular-endothelial growth factor receptor 2) to identify putative endothelial progenitor cells (EPCs) [6, 7]. The numbers of PCs are reduced in patients with cardiovascular disease (CVD) and PAD [5, 8–12], including SLI [13]. Moreover, numbers of PCs circulating in peripheral blood (PB PCs) are inversely related to cardiovascular outcomes and may be used as a biomarker to predict adverse outcomes [14–17]. These findings are thought to indicate that impaired PC driven vascular repair plays a role in CVD pathogenesis.

Prior to mobilization into the circulation, CD34^+^ or CD133^+^ PCs reside in the bone marrow (BM) niche [18]. The reduction in PB PC numbers is caused by a complex and multifactorial mechanism, involving both a defect in PC mobilization and depletion of the BM reserve. For instance, patients with diabetes have been shown to display a ‘mobilopathy,’ i.e., they respond less to exogenously administered mobilizing factors such as Granulocyte Colony-Stimulating Factor (G-CSF) or Stromal Derived Factor (SDF) 1a [19, 20]. In contrast, a study in PAD patients showed that patients with more severe PAD mobilized more PCs to the peripheral circulation in response to an ischemic stimulus. There are also indications that CVD patients in general have lower BM PC numbers [21] and that reduced numbers of PCs in circulation may just be a proportional reflection of the BM content. In a previous cross-sectional study in SLI patients, we have shown that PC numbers are reduced in the BM itself [13]. Other studies have shown that higher BM PC numbers are associated with recovery after myocardial infarction, but these studies do not report on PB PC numbers [22, 23]. Thus, currently it is unclear whether insufficient mobilization or an absolute deficiency of PCs is predominant, and how this relates to CVD progression.

In parallel, therapies to increase the number of progenitor cells in circulation have been developed, aiming to augment vascular repair in acute or ischemic vascular disease.

While pre-clinical studies and early clinical trials showed promising results, later trials with more rigorous design did not show CD34^+^-based therapy to be more effective than placebo. This further underscores the necessity of elucidating the link between PB and BM PCs and outcomes.

In the present study, we examine the relationship between PB and BM PCs, and Amputation-Free Survival (AFS) in SLI patients. Furthermore, we attempt to separate potential BM reserve exhaustion from a mobilization defect in determining AFS risk. To this end, we use data obtained in the JUVENTAS trial, a randomized controlled trial investigating autologous BM cell therapy for the treatment of SLI [24], for which we have extended the initial follow-up.

## Materials and methods

### Study population

The study population of this prospective cohort study consisted of patients included in the JUVENTAS trial; a single-center double-blind randomized placebo-controlled trial investigating repetitive intra-arterial infusion of autologous BM mononuclear cells (BMMNCs) for the treatment of SLI. Trial results of the primary analysis have been published elsewhere [24]; study inclusion was from September 2006 until June 2012. Inclusion criteria for the trial were severe infra-popliteal atherosclerosis, Fontaine grade IIB–IV, and ineligibility for surgical intervention. Exclusion criteria were a history of neoplasm or malignancy in the past 10 years, concomitant disease with life expectancy of less than 1 year, inability to obtain sufficient BM aspirate, known infection with human immunodeficiency virus, hepatitis B or C virus, and an inability to complete follow-up. Patients were randomized 1:1 to receive 3 intra-arterial infusions of autologous BMMNCs or placebo into the common femoral artery of the affected limb. The primary outcome was the incidence of major amputation, defined as amputation through or above the ankle joint, at 6 months after inclusion. For the present study, we have extended follow-up until the beginning of 2015, using AFS as primary outcome. In addition to the study population, 17 healthy controls were included, who underwent BM aspiration under full anesthesia prior to elective major surgery.

### Ethics statement

The study has been approved by the Medical Ethics Committee of the UMC Utrecht (no. 06/030) and was conducted in accordance with the declaration of Helsinki. All included patients provided written and verbal informed consent prior to inclusion.

### Sample preparation

Prior to treatment allocation, approximately 100 ml of BM was harvested from the right iliac crest by an experienced hematologist under local anesthesia and conscious sedation. Peripheral blood (PB) samples were obtained by venipuncture of the antecubital vein. Flow cytometry on PB and BM was performed using lyse-and-wash protocols. 100 µl of PB or BM was incubated with the following antibody panels: 1: anti-CD34-FITC (BD Pharmingen, San Diego, CA, USA), anti-KDR-PE (R&D Systems, Minneapolis, MN, USA); 2: anti-CD133-PE (Miltenyi Biotec, Bergisch Gladbach, Germany); 3: anti-CD184-PE (BD Pharmingen) and anti-CD14-ECD (Immunotech, Coulter, France), 4: anti CD-105-FITC (R&D) and anti-CD14-ECD; 5: anti-CD140b-PE (BD Pharmingen), for 45 min in the dark. Erythrocytes were lysed in an ammonium chloride buffer and remaining cells were washed with PBS and analyzed by flow cytometry (FC 500, Beckman Coulter, Fullerton, CA, USA). All samples were processed in duplicate. Spectral compensation was performed by using IgG isotype controls for the relevant fluorophores. Flow Cytometry Data Analysis Data were analyzed using FlowJo software version 10.0.8 (Treestar Inc., Ashland, OR); all files were analyzed by a blinded assessor. See supplementary methods and Supplementary Fig. 1 for a description of the gating strategy. To account for variations in acquisition occurring due to inconsistency in erythrocyte lysis, all cell numbers are corrected for the number of granulocytes, as this population can be most reliably identified on FSC/SSC. Colony Forming Units Myeloid precursor (colony forming unit-granulocytes and monocytes; CFU-GM) and erythroid precursors (burst forming unit-erythroid; BFU-e) forming capacity assays of the BMMNC were performed using Methocult H4534 and H4434 (Stemcell Technologies Inc, Vancouver, BC, Canada), respectively, in accordance with the manufacturer’s instructions.

### Statistical analysis

Continuous variables are presented as means ± standard deviation (SD) or as medians with Interquartile Range (IQR). Differences between patients who reached an endpoint and who did not were assessed by chi-square test for categorical variables, student’s *t* test for normally distributed continuous, and a Mann–Whitney *U* test for non-normally distributed variables. Cell counts were non-normally distributed and were transformed by taking the natural logarithm (e) of the (cell number + 1). Correlations between cell types were quantified by spearman rank coefficient (*ρ*). To assess the relative contribution of mobilization, patients were divided into quadrants of either high and low numbers of BM and PB PCs, based on the median values of PC numbers. Survival analysis was performed using Cox proportional hazards regression models; throughout this paper Hazard Rates (HR) are given as exp(*β*), where *β* is the slope of the regression model for one *e*-fold increase in PCs. In cases where regressor distribution was not known, a restricted cubic spline was fit in order to obtain an unbiased fit. Covariates to Cox regression were added as described in the relevant tables. For multivariable models, automated backward exclusion of model factors based on an AIC criterion was performed in order to derive an optimal model. The proportional hazards assumption was evaluated by plotting Schoenfeld residuals. An a priori power analysis assuming a standardized *β* of 1.87 as in Fadini et al. [17] yielded a power of > 0.99 for PB CD34 PCs. *p* Values < 0.05 were considered statistically significant. The Benjamini–Hochberg method was used to limit the False Discovery Rate resulting from multiple testing in parallel evaluation of cell types. All analyses were performed in the R computing environment, version 3.1.0. [25].

## Results

### Trial and patients

One hundred and sixty patients were included in the JUVENTAS trial and were followed for a median of 3.0 years at the point of the present analysis, totaling 509 patient-years of follow-up. Table 1 presents characteristics of the trial population, divided into patients with and without a major event (defined as amputation or death) during follow-up. Patients who underwent amputation or died were more likely to be male, older, and to have had a history of cerebrovascular accident (CVA) or myocardial infarction. Moreover, they were more likely to have a more advanced disease stage and larger ulcer size. As we reported previously, BMMNC treatment did not affect AFS. The number of CD34^+^ cells in the administered graft did not correlate with any outcome parameter [24].

**Table 1:** Baseline table: table displaying baseline characteristics of the full cohort, patients who underwent an event and patients who did not undergo an event

Juventas baseline	Total cohort (*n* = 160)	Event (*n* = 67)	No event (*n* = 93)	*p* value
Sex (M/F)	108/52 (68%)	52/15 (78%)	56/37 (58%)	**0.03**
Age (years)	67 [56–76]	71 [62–79]	62 [52–72]	**0.0012**
BMI (kg/m^2^)	26.4 (4.52)	26.6 (4.96)	26.3 (4.20)	0.74
Smoking (current/past/never)	42/95/23	13/44/10	29/51/13	0.24
Systolic BP (mmHg)	131.0 (19.5)	132.5 (20.57)	130.0 (18.6)	0.42
Diastolic BP (mmHg)	72.9 (9.9)	72.1 (10.22)	73.5 (9.7)	0.39
Creatinine (µmol/l)	90.0 [75–115]	105 [75–147]	87 [75–107]	**0.03**
GFR (MDRD, ml/min/1.73 cm^2^)	69.7 (27.6)	66.1 (32.0)	72.3 (23.8)	0.18
Cholesterol (mmol/l)	4.26 (1.14)	4.11 (1.13)	4.37 (1.13)	0.15
HDL (mmol/l)	1.20 (0.42)	1.11 (0.47)	1.26 (0.39)	**0.022**
Triglycerides (mmol/l)	1.66 (1.01)	1.71 (1.12)	1.62 (0.94)	0.58
hsCRP (mg/ml)	5.50 [2.1–13.9]	7.65 [3.0–13.9]	4.37 [1.8–11.4]	**0.006**
DM (IDDM/NIDDM/none)	33/27/100	16/14/37	17/13/63	0.27
History of CVA	23 (14%)	19 (28%)	4 (4%)	**0.00005**
History of MI or Angina	66 (41%)	36 (46%)	30 (32%)	**0.01**
History of dialysis	5 (3.1%)	2 (3.0%)	3 (3.2%)	0.99
Anti-platelet drugs	112 (70%)	49 (73%)	63 (68%)	0.58
Oral anti-coagulants	61 (38%)	26 (39%)	35 (38%)	0.99
Lipid-lowering drugs	135 (84%)	55 (82%)	80 (86%)	0.65
ACE inhibitor	62 (39%)	29 (43%)	33 (35%)	0.4
ß-Blocker	71 (44%)	35 (52%)	36 (39%)	0.12
Diuretics	72 (45%)	36 (54%)	36 (39%)	0.08
Rutherford class (3/4/5/6)	8/51/92/9	0/16/45/6	8/35/47/3	**0.007**
Fontaine class (IIB, III, IV)	8/51/101	0/16/51	8/35/50	**0.004**
Ulcer	101 (63%)	51 (76%)	50 (54%)	**0.006**
Ulcer Area (cm^2^)	1.63 (1.0–4.3)	2.13 [1.0–5.0]	1.50 [0.75–2.75]	**0.04**
BMMNC treatment	81 (51%)	33 (49%)	48 (52%)	0.89

Bold values indicate *P* = <0.05

Numbers between parentheses indicate standard deviation (SD) and numbers between square brackets indicate interquartile range [IQR]

### Blood and bone marrow progenitor cell associations with amputation-free survival

**Fig. 1:**
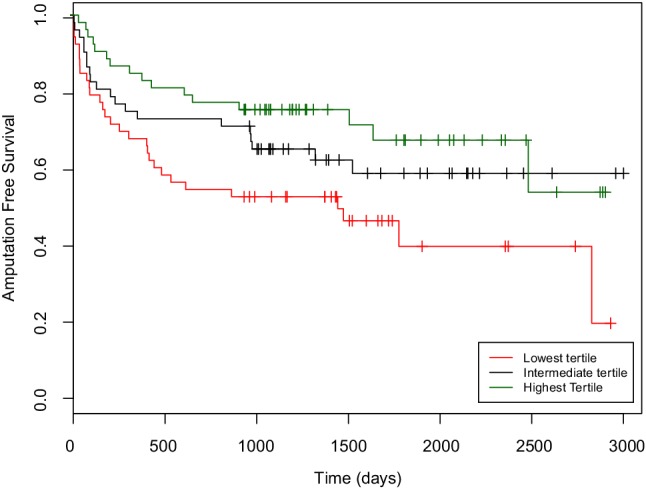
Kaplan–Meier curve for amputation-free survival as divided by tertiles of circulating CD34^+^ progenitor cells in peripheral blood

The univariate association of PB and BM subpopulations with AFS is presented in Table 2. Higher numbers of CD34^+^ and CD133^+^ PCs in PB were associated with longer AFS (Both a Hazard Ratio (HR) of 0.56, *p* = 0.003 and *p* = 0.0007, respectively) per *e*-fold increase in number of PCs. Division into tertiles showed a HR of 2.02 of the lowest compared to the highest tertile for PB CD34^+^ PCs (Fig. 1). PB CD34^+^ and CD133^+^ showed modest potential as biomarkers in this study, with C-statistics on the ROC curve of 62.1% and 63.8%, respectively (Supplemental Fig. 3). We observed no association between CD34^+^/KDR^+^ cells and AFS (HR 0.93, *p* = 0.55). There was a trend towards an association between CXCR4^+^ cells and major outcomes (HR 0.77, *p* = 0.049), but this may be due to chance, considering the multiple hypotheses tested. The other cell populations assessed, CD14^+^ monocytes and CD140b (PDGFRb)^+^ cells, were not associated with AFS. CD105^+^ mesenchymal cells were not detectable in PB.

**Table 2:** Cell populations and amputation-free survival: table showing hazard ratios (HR) for amputation or death associated with various (progenitor) cell populations in PB and BM as measured by flow cytometry

Cell population	Blood			Adj *p* value	Bone Marrow			Adj *p* value
	HR	95% CI	*p* value		HR	95% CI	*p* value	
CD34^+^	0.56	0.38–0.83	**0.003**	**0.012**	0.58	0.39–0.87	**0.008**	**0.03**
CD34^+^/KDR^+^	0.93	0.74–1.17	0.55	0.99	0.85	0.63–1.16	0.3	0.99
CD133^+^	0.56	0.40–0.80	**0.0007**	**0.003**	0.53	0.30–0.93	**0.028**	0.12
CD14^+^	0.81	0.52–1.24	0.33	0.99	1.27	0.70–2.28	0.433	0.99
CD140b^+^ (PDGFRb)	0.98	0.82–1.19	0.89	0.99	0.87	0.6–1.27	0.48	0.99
CD105^+^	N/A	N/A	N/A	N/A	0.91	0.66–1.26	0.58	0.99
CXCR4^+^	0.77	0.60–0.99	**0.049**	0.21	0.78	0.54–1.12	0.18	0.76
CFU-GM	N/A	N/A	N/A	N/A	0.55	0.36–0.87	**0.01**	**0.04**
BFU-E	N/A	N/A	N/A	N/A	0.62	0.37–1.04	0.07	0.27

Bold values indicate *P* = <0.05

For each cell population, HR and 95% confidence interval is given, as well as associated *p* value and a *p* value adjusted for multiple testing

BM PC populations showed a highly similar risk profile compared to PB PCs. Higher numbers of CD34^+^ and CD133^+^ were associated with longer AFS (HR of 0.58, *p* = 0.008 and HR of 0.53, *p* = 0.028, respectively). These results were confirmed by colony-forming assays for granulocytes/monocytes (CFU-GM, HR 0.55, *p* = 0.01); the colony-forming assay for burst forming erythroid units showed a trend towards association with AFS (HR 0.62, *p* = 0.07). Other BM subpopulations were not associated with AFS (Table 2).

Analysis of the separate components of the composite outcome—amputation and death—showed a similar trend in associations with BM and PB progenitor cells (Supplementary Table 1).

### Correlations between blood and bone marrow PC numbers

The numbers of PCs identified by the two markers CD34 and CD133 in PB and BM were highly correlated (*r* = 0.72 in PB and *r* = 0.80 in BM, Supplemental Fig. 2). Correlations of the numbers of cells displaying either marker between PB and BM were intermediate (*r* = 0.40 and 0.31 for CD34^+^ and CD133^+^ cells, respectively), with absolute numbers of PCs being approximately 20-fold higher in BM aspirates. CD34^+^/KDR^+^ double-positive cells correlated poorly with other PCs and there was no correlation between PB and BM.

Coefficients of variance (CV) were also higher for CD34^+^/KDR^+^ cells (21%) compared to other PC cell types (~ 10%), indicating an increased amount of random fluctuation in the assay.

Both CD34^+^ and CD133^+^  BM PC numbers were correlated to CFU-GM (*r* ≈ 0.44).

### Relative contribution of blood and bone marrow progenitor cell numbers to amputation-free survival

**Fig. 2:**
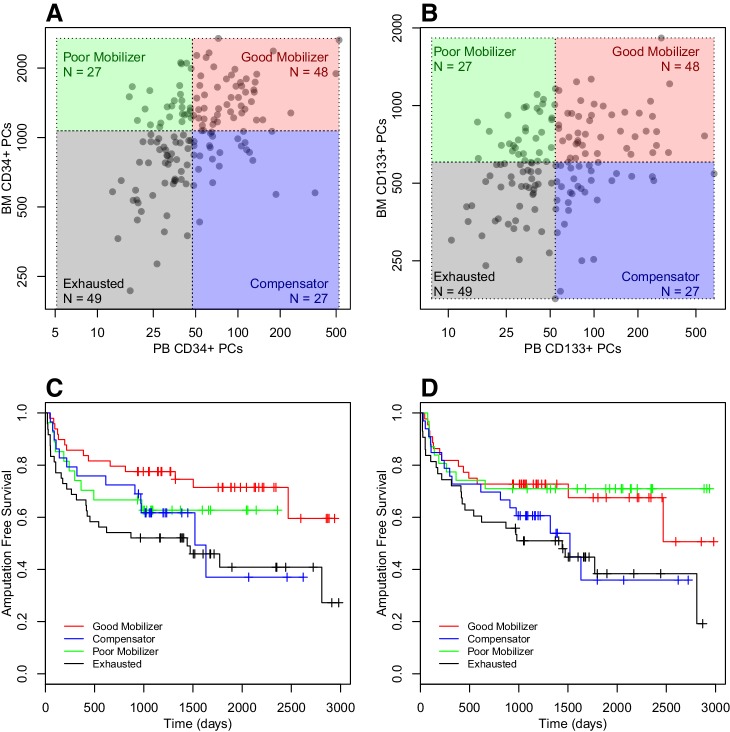
Relative contribution of PB and BM PCs to cardiovascular risk: patients were divided into quadrants based on whether PB or BM values were higher or lower as compared to the median for either cell population. Division into quadrants is shown for CD34^+^ cells in (**a**) and for CD133^+^ cells in (**b**). The quadrant of patients with both BM and PB PC numbers below the median was designated “Exhausted”; the quadrant with PB PC numbers below the median, but BM PC numbers above the median, was designated “Poor Mobilizers”; the quadrant with PB PC numbers above the median was designated “Compensators”; and the quadrant with both high PB and BM PC numbers was called “Good Mobilizers.” **c** and **d** The Kaplan Meier Survival curves for the four quadrants of CD34^+^ PCs and CD133^+^ PCs, respectively

To attain insight in the relative contribution of BM exhaustion and a mobilization defect towards risk of amputation and death, we examined the variation around the identity line for PB versus BM PCs. Analogous to a study by Fadini et al [26], we divided patients into four quadrants, on the basis of high and low numbers in PB versus BM (Fig. 2a, b). Patients, who showed low numbers of PCs in both PB and BM, were defined as patients with a depleted BM reserve and termed “Depleted.” Patients with high numbers of PCs in BM, but not in PB, were considered to possess a relatively isolated mobilization defect and were termed ‘Poor Mobilizer.’ Patients who have low numbers of PCs in BM, but relatively high numbers of PCs in PB, were thought to display active signs of compensation and were designated “Compensators.” Patients with high number of PCs in both PB and BM were termed “Good Mobilizers.” Kaplan Meier survival curves for the four quadrants are shown in Fig. 2c, d. For both CD34^+^ and CD133^+^ PCs, the “Exhausted” group displayed the worst prognosis (HR 2.5, *p* = 0.005 and HR 2.1, *p* = 0.03 for CD34^+^ and CD133^+^ PCs, compared to “Good Mobilizers”). The “Poor Mobilizers” showed a lower risk for AFS than the “Compensators” (HR 1.6 vs. 1.9 for CD34^+^ PCs and HR 0.84 vs. 1.7 for CD133^+^ PCs).

In a comparison of the JUVENTAS cohort to a small cohort of 17 healthy BM donors, we observed that healthy controls displayed a 1.88-fold higher number of PB CD34^+^ PCs (*p* = 0.003) but a 2.25-fold higher number of BM CD34^+^  PCs (*p* = 0.00002). In 6/17 cases, the number of BM CD34^+^ PCs exceeded the range of observed values in SLI patients (Supplementary Fig. 4). Numbers of PB CD133^+^ PCs were 1.13-fold higher in healthy controls (*p* = 0.6), but BM CD133^+^ PCs numbers were 1.7-fold higher (*p* = 0.003).

In addition to the quadrant method, we examined whether a mobilization index, the ratio between PB/BM PCs, was associated with major events. The mobilization index of neither CD34^+^ cells nor CD133^+^ was associated with AFS (Supplementary Fig. 5).

### Association of progenitor cell numbers with traditional risk factors

Associations between the number of PB and BM PCs and traditional cardiovascular risk factors are presented in Table 3. Most PC populations were slightly lower in males, except for CD133^+^ cells in PB, which were nearly 9% lower. PC numbers in both blood and BM were negatively correlated with age, although the effect was greater in blood (*r* = − 0.24 and − 0.19 for CD34^+^ and CD133^+^ PCs compared to *r* = − 0.14). There was a significant association of PC numbers with Glomerular Filtration Rate (GFR), which was of similar magnitude (*r* ~ 0.2) in both blood and BM. Total cholesterol and triglyceride levels were both positively correlated with PB PC numbers. There was an inverse association between more advanced stages of SLI (Fontaine IV vs. IIb and III) and PC numbers.

**Table 3:** PCs and Risk Factors: Association of cardiovascular risk factors and numbers of progenitor cells in PB and BM

Risk factor	PB CD34		PB CD133		BM CD34		BM CD133	
	Effect	*p* value	Effect	*p* value	Effect	*p* value	Effect	*p* value
Sex (male)	− 2.2%	0.47	− 8.8%	0.005	− 1.9%	0.13	− 1.2%	0.24
Age (years)	*ρ* − 0.24	**0.002**	*ρ* − 0.19	**0.015**	*ρ* − 0.14	0.07	*ρ* − 0.14	0.08
BMI (kg/m^2^)	*ρ* 0.05	0.51	*ρ* − 0.01	0.89	*ρ* 0.04	0.65	*ρ* − 0.06	0.45
Smoking (ever)	5.9%	0.17	1.3%	0.77	1.3%	0.46	1.2%	0.41
Systolic BP (mmHg)	*ρ* − 0.01	0.90	*ρ* − 0.01	0.88	*ρ* 0.06	0.46	*ρ* 0.03	0.72
Diastolic BP (mmHg)	*ρ* 0.10	0.20	*ρ* 0.07	0.41	*ρ* 0.05	0.53	*ρ* 0.05	0.54
GFR (MDRD, ml/min/1.73 cm^2^)	*ρ* 0.23	**0.003**	*ρ* 0.18	**0.022**	*ρ* 0.23	**0.004**	*ρ* 0.2	**0.012**
Cholesterol (mmol/l)	*ρ* 0.22	**0.005**	*ρ* 0.16	**0.046**	*ρ* 0.17	**0.03**	*ρ* 0.14	0.08
HDL (mmol/l)	*ρ* 0.10	0.23	*ρ* 0.08	0.29	*ρ* 0.14	0.08	*ρ* 0.12	0.14
hsCRP (mg/l)	*ρ* − 0.33	< **0.001**	*ρ* − 0.31	< **0.001**	*ρ* − 0.35	< **0.001**	*ρ* − 0.31	< **0.001**
Triglycerides (mmol/l)	*ρ* 0.20	**0.011**	*ρ* 0.17	**0.033**	*ρ* 0.03	0.67	*ρ* 0.01	0.92
Diabetes mellitus	− 2.3%	0.42	− 4.3%	0.18	− 2.0%	0.11	− 1.3%	0.23
History of CVA	− 1.4%	0.72	− 2.0%	0.65	− 0.3%	0.85	0.2%	0.86
History of MI or angina	− 4.3%	0.14	− 4.3%	0.17	− 3.1%	**0.009**	− 2.6%	**0.013**
History of dialysis	− 20.0%	**0.04**	− 17%	0.10	− 10%	**0.006**	− 7.6%	0.01
Anti-platelet drugs	1.1%	0.73	0.1%	0.98	− 0.3%	0.80	− 0.6%	0.62
Oral anti-coagulants	− 2.3%	0.44	1.7%	0.61	− 0.8%	0.51	0.0%	0.97
Lipid-lowering drugs	− 1.3%	0.73	− 2.3%	0.59	− 0.7%	0.68	− 0.2%	0.92
ACE inhibitor	0.9%	0.77	− 1.0%	0.75	2.1%	0.09	− 1.4%	0.21
ß-Blocker	0.8%	0.78	1.6%	0.63	− 0.2%	0.89	− 0.3%	0.81
Diuretics	− 3.1%	0.27	− 6.7%	**0.03**	− 2.0%	0.09	− 1.2%	0.26
Fontaine IV (ulcer or necrosis)	− 10.0%	**0.0003**	− 8.0%	**0.009**	− 2.1%	0.09	− 3.0%	**0.004**
Ulcer area (cm^2^)	*ρ* − 0.15	0.13	*ρ* − 0.17	0.09	*ρ* − 0.15	0.14	*ρ* − 0.09	0.40

Bold values indicate *P* = <0.05

For each cell population, an estimate of effect size is given: % increase or decline for binary variables and Spearman’s *ρ* for continuous variables

### Adjustment for traditional risk factors

Adjusted models, correcting for sex, age, GFR, history of CVA, history of MI or Angina, presence of ulcers, higher triglyceride levels, and cholesterol levels, show higher HRs for PB PC numbers than unadjusted models (CD34^+^: HR = 0.71, *p* = 0.07, CD133^+^: HR = 0.64, *p* = 0.012). In BM, the adjusted HR was 0.49 (*p* = 0.03) for CD34^+^ PCs, and 0.65 (*p* = 0.16) for CD133^+^ PCs. Prior history of CVA was most strongly associated with amputation or death, followed by disease stage (presence of ischemic ulcers). In all models, both PB and BM PCs proved to be independent predictors of major outcomes. For further details regarding adjusted and reduced models, see Supplemental Table 2.

## Discussion

The present study shows that CD34^+^ and CD133^+^ PCs in both PB and BM were associated with AFS in patients with SLI independent of traditional risk factors. The respective BM and PB PC populations displayed similar risk profiles and were moderately interrelated. Subdivision of patients based on their relative PB and BM PC values, separating patients with BM depletion from those with a mobilization defect, showed that BM PC numbers more strongly determined outcome compared to PB PC levels. This suggests that the impairment in endogenous vascular repair mechanisms in SLI is for a large part due to a lower availability of PCs in the BM, rather than solely due to a defective mobilization to the PB.

There are few prospective studies that have examined the link between PB PCs and cardiovascular outcomes [14–17]. To our knowledge, this is the first study that investigates the relationship between BM PCs and AFS in SLI. Schmidt-Lucke et al. [15] and Werner et al. [16] were the first to show that CD34^+^/KDR^+^ PB EPCs were associated with cardiovascular outcomes in patients with coronary artery disease. More recent studies also showed that CD34^+^ PB PCs were associated with myocardial infarction and death in patients undergoing coronary angiography [14] and patients with Type II Diabetes [17, 27]. HRs for major outcomes with regard to CD34^+^ PCs observed in those studies were of similar magnitude as the ones observed in the present study, with roughly a halving of risk per *e-*fold increase or 1 SD increase, depending on reporting of results. In the present study, we did not observe an association between CD34^+^/KDR^+^ cells and AFS. This is in agreement with the study of Patel et al. [14], who also did not find an association of CD34^+^/KDR^+^ cells with cardiovascular events in two large cohorts comprising a total of 905 patients. The difference with the earlier studies might lie in the anti-KDR antibody used, as currently available monoclonal anti-KDR antibodies show poor reproducibility in staining [28].

Very few studies have been able to investigate BM samples in patients with CVD, and this is the first longitudinal study that examines whether BM PCs predict future cardiovascular events. Prior cross-sectional studies have shown a reduction in CFU-GM and CD34^+^ BM PCs in smaller cohorts with chronic ischemic heart disease. A study by Schutt et al. [22] examined prognostic implications of lower BM PC numbers after acute myocardial infarction, but found no association of BM PCs with infarct size. However, the same group showed that in patients with chronic ischemic heart disease [23], which may have more similarity to chronic ischemic SLI patients, extremely high or low (> 2 SD) numbers of CD34 ^+^ PCs in BM were associated with improvement or decrease of left ventricular ejection fraction (LVEF), respectively, in the course of disease.

One of the most interesting aspects of the present study is that both BM and PB PC numbers were quantified, which may provide insight in the underlying mechanism behind the reduction of PC numbers. It has classically been thought that lower numbers of PB PCs reflect a defect in mobilization from the BM, due to desensitization to or reduced availability of signaling molecules such as nitric oxide [29]. Indeed, patients suffering from CVD, particularly diabetes, are resistant to exogenous PC mobilizing factors such as G-CSF [30]. On the other hand, more recent studies also show a reduction of PC numbers in BM of patients with CVD [13, 31, 32].

In the present study, we show that there is a moderate correlation between PB and BM numbers (*R* ~ 0.4 for CD34^+^ cells), which is in agreement with previous studies (Fadini et al. *R* ~ 0.43 and Cogle et al. *R* ~ 0.34 [23]). This implies that common factors affect both BM and PB PC numbers: in this study, we observed that disease history, age, and renal function were associated with PC numbers regardless of compartment. As the correlation mentioned above only explains a small proportion of the variance, we have used the reasoning as proposed by Fadini et al. [26], and subdivided patients based on relative PB-to-BM numbers. In this study, we are the first to show the prospective implications of a relative alteration in BM and PB PC numbers. We show that patients who appear to have an isolated mobilization defect, i.e., high numbers of BM PCs but low numbers of PB PCs (“Poor Mobilizers”) have a fairly benign risk profile, at least as good as patients who have high PB PC numbers but low BM PC numbers (“Compensators”), and nearly as good as the “Good Mobilizers.” Furthermore, CD34^+^ and CD133^+^ are comparatively higher in BM of healthy donors compared to controls than the respective PB PC subpopulations. Taken together, our data suggest that in the two competing visions for the etiology of the reduction in PB PC numbers observed in CVD mobilization defect versus BM exhaustion—the latter has an important if not dominant role.

Other cell populations were not associated with AFS in our study. Notably, we did not find an association between CD14^+^ cells and AFS, even though previous studies have shown that various monocyte subsets are associated with cardiovascular events [33, 34]. However, we did not differentiate between classical and non-classical or intermediate monocytes, which may explain the negative finding [33]. We observed a trend towards an association with PB CXCR4^+^ cells and AFS, which proved not robust enough for multiple testing and no trend in BM CXCR4^+^ cells. A recent report found that BM CXCR4^+^ cells are associated with functional improvements over time in patients with ischemic heart disease [20].

Lastly, we did not observe associations with AFS and numbers of BM cells positive for CD105^+^, a marker for Mesenchymal Stromal Cells (MSCs) [35]. MSCs constitute the perivascular part of the hematopoietic niche and are not mobilized into the circulation [36]; congruently we did not observe CD105^+^ cells in PB. The lack of association between CD105^+^ cell numbers in BM and AFS or disease severity is in agreement with a previous study with culture-expanded MSCs in this cohort [37], in which we observed that SLI MSCs did not differ from controls.

The question remains how the link between PC numbers and cardiovascular outcomes should be interpreted. Had the results shown that the number of (mobilized) cells in circulation determined risk of AFS, this would have supported the hypothesis of a causal role of circulating progenitor cells in vascular repair. The data show, however, that circulating cell numbers and mobilization are of minor importance (if not a nuisance variable) compared to PC numbers in BM. The most parsimonious interpretation is therefore that a lower number of BM and consequently PB CD34^+^ cells indicate the general disease state which also predisposes to CVD. A plausible explanation may be that lower progenitor cell numbers reflect increased senescence on a cellular level and frailty on the level of the patient. It has been shown that CVD patients generally have shorter telomeres than healthy controls, which has been associated with lower CD34^+^ numbers [38, 39]. Alternatively, the reduced number of PCs can be a result of a chronic inflammatory state, which has deleterious effects on both cardiovascular risk and BM PC numbers [40, 41].

Lastly, the reduced PC numbers might be closely related to the outcome of vascular disease itself. In a previous study, we have shown that CLI patients have decreased BM vascularization and innervation, which might also affect the PC compartment [42]. Similar results have been observed in animal studies, demonstrating a relationship between reduced innervation and reduced PC proliferation [43].

The findings presented in this study may also have a bearing on the rationale of BM MNC therapy in cardiovascular disease, and SLI in particular. Autologous cell therapy using BMMNCs or BM PCs is increasingly proposed as a treatment for SLI patients [44], based on the reasoning that a mobilization defect can be circumvented by BM aspiration. If the BM PC population is also reduced, however, the efficacy of autologous therapy may be limited as BM PC numbers are linked to pro-angiogenic effects of BM MNC isolates [45]. Two studies indeed report that non-responders to PC administration as treatment for PAD received lower numbers of PC [46, 47]. However, in the JUVENTAS cohort, total administered BM PC numbers were not associated with better outcomes [24]. Additionally, another study showed that BM PC proliferative capacity rather than numbers was associated with neovascularization [48]. This further underscores that lower BM PC numbers are symptomatic of functional BM alterations. Furthermore, our data suggest that the primary rationale of inducing PC mobilization to ameliorate cardiovascular disease may be flawed. This may also explain the persistent negative results of attempts to improve cardiovascular outcomes using Granulocyte Colony-Stimulating Factor (G-CSF) to promote mobilization of progenitor cells [49].

In summary, we have demonstrated that lower numbers of both PB and BM PCs are associated with a worse prognosis in SLI patients. Moreover, BM PC numbers correlated with PB PCs and show a similar risk profile with regard to cardiovascular outcomes. Our findings imply that a depletion of the BM niche, rather than a defect in PC mobilization, underlies the association between PB PC numbers and cardiovascular risk.

## Electronic supplementary material

Below is the link to the electronic supplementary material.

Supplementary material 1 (PDF 492 KB)
