# Supplementary material for: Exhaustion of the bone marrow progenitor cell reserve is associated with major events in severe limb ischemia
Source: Angiogenesis. 2019 Mar 30;22(3):411–20. doi: 10.1007/s10456-019-09666-0 (PMC6652783; doi:10.1007/s10456-019-09666-0)
Supplement: Supplementary file 1 — Supplementary material 1 (PDF 492 KB) [file 10456_2019_9666_MOESM1_ESM.pdf]

Supplementary Figures and Tables

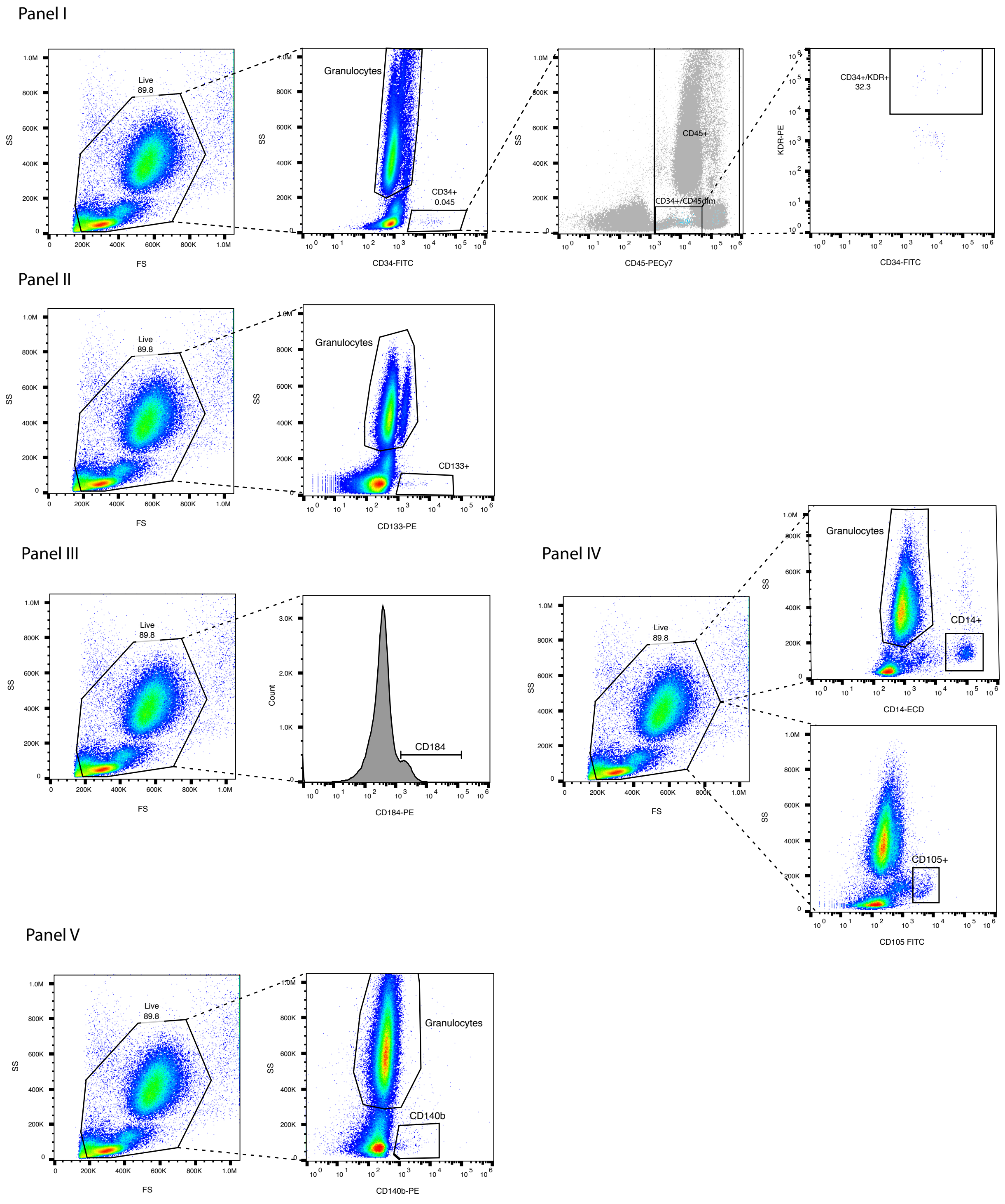

Figure 1: **Gating Strategy:** Figure showing the gating strategies for the 6 flow cytometry panels as described in the *Methods* section. Data analysis was performed by first creating a 'Live'-gate around the three populations of lymphocytes, monocytes and granulocytes on the Forward/Sideward-Scatter plot (see suppl fig 1). For *panel I*: **CD34+** were first gated and the population was further refined by selecting the **CD45dim** subpopulation. Within the latter population the **KDR+** cells were subsequently enumerated (see suppl fig 1). In *panel II*, **CD133+** cells were selected by plotting side-scatter versus PE and gating CD133+ cells in the lymphocytic region. In *panel III* the the **CXCR4/CD184+** cells were gated on the histogram plot. In *panel IV* **CD14+** and **CD105+** cells were gated as shown, no double positive cells were observed. In *panel V* **CD140b/PDGFRb+** Cells were gated in the lymphocytic region.

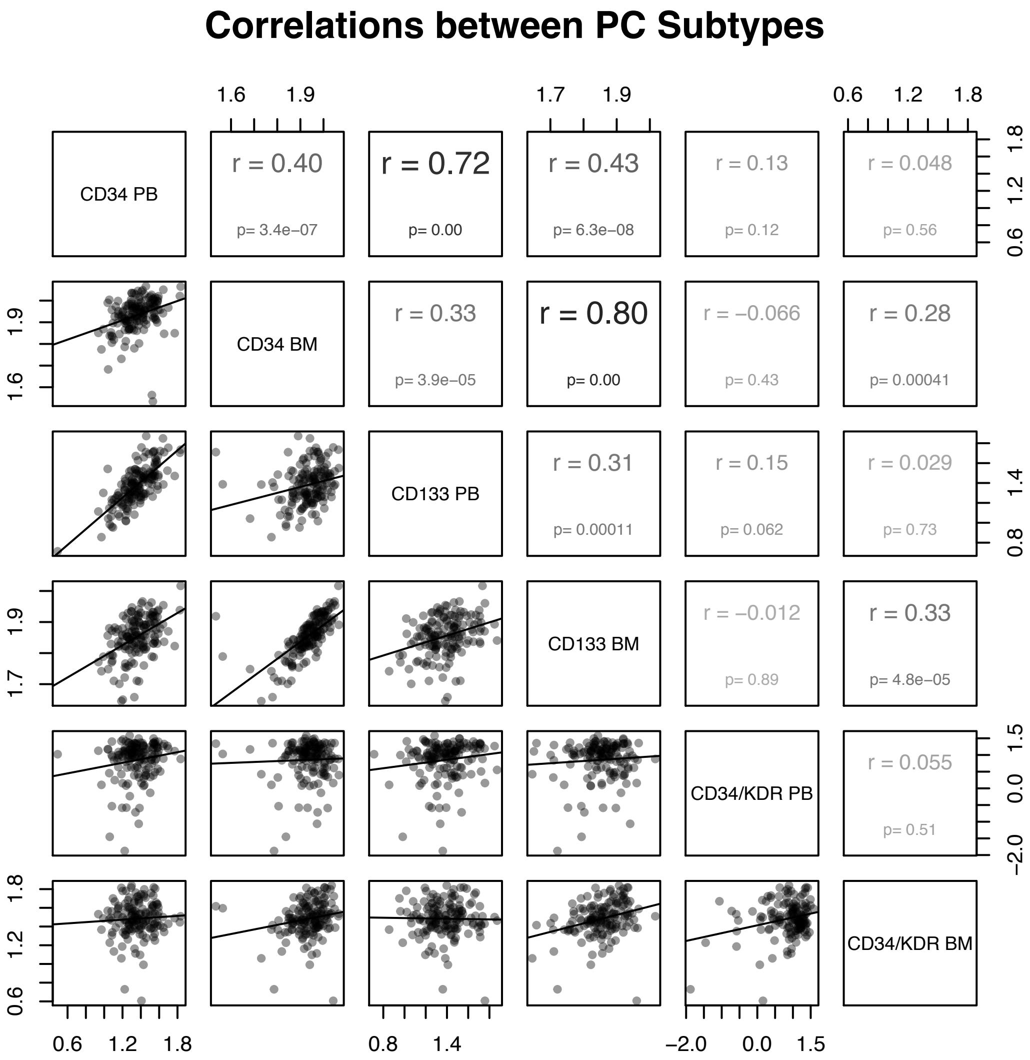

Figure 2: **Correlationmatrix:** Correlations between PC populations in peripheral blood and bone marrow. In the left/lower half correlation plots are given, in the right/upper half the Spearman's rank correlation coefficent for an pairwise comparison and the associated p-value are given.

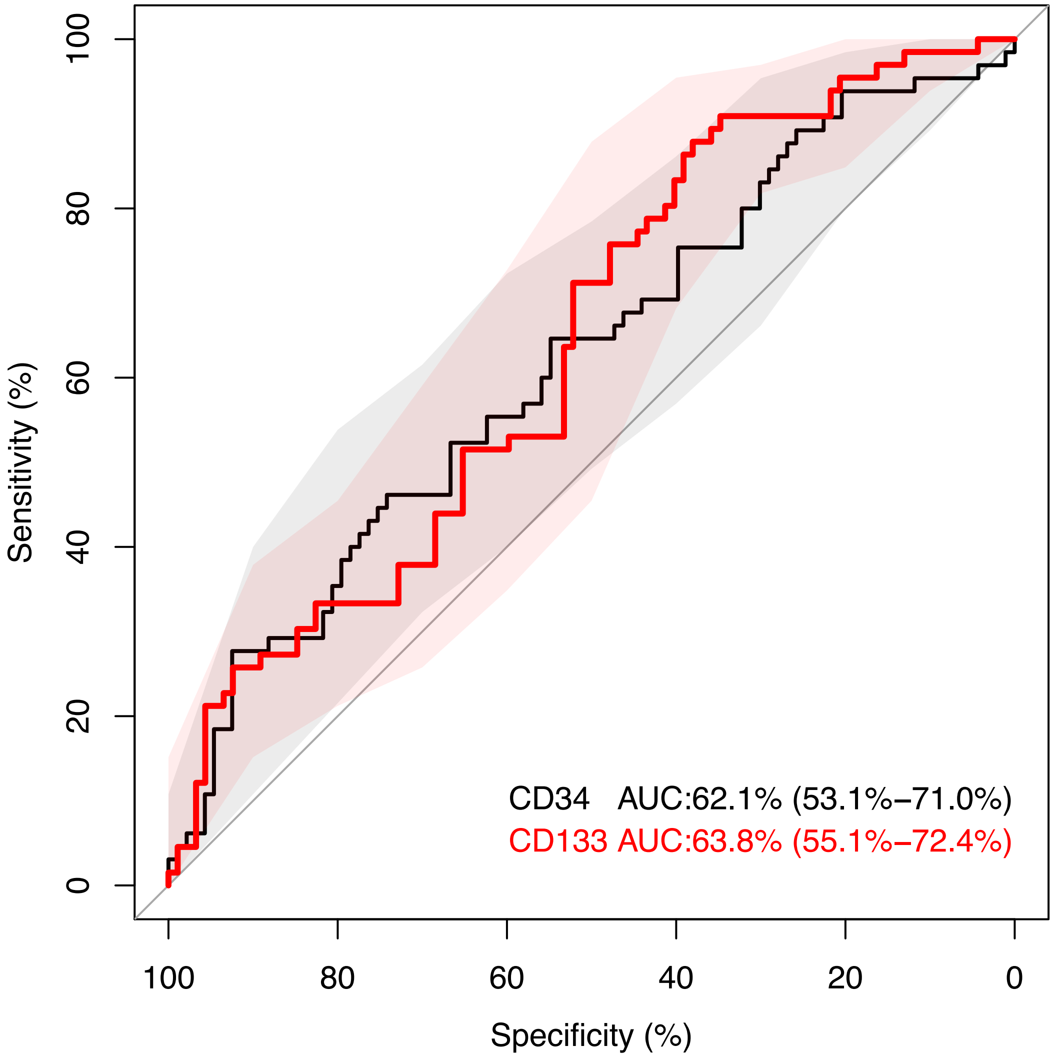

Figure 3: **ROC Curve:** Receiver Operated Curve (ROC) displaying the sensitivity and specificity of various cutoffs in PC numbers in discrimnating patients who will undergo an event and patients who will not experience a major event. Areas Under Curve (AUCs) + 95% CI for CD34+ (in black) and CD133+ (in red) cells in peripheral blood are given in the lower right corner. Shaded areas indicate 95% confidence intervals of the curves, as derived by bootstrapping (2000 iterations).

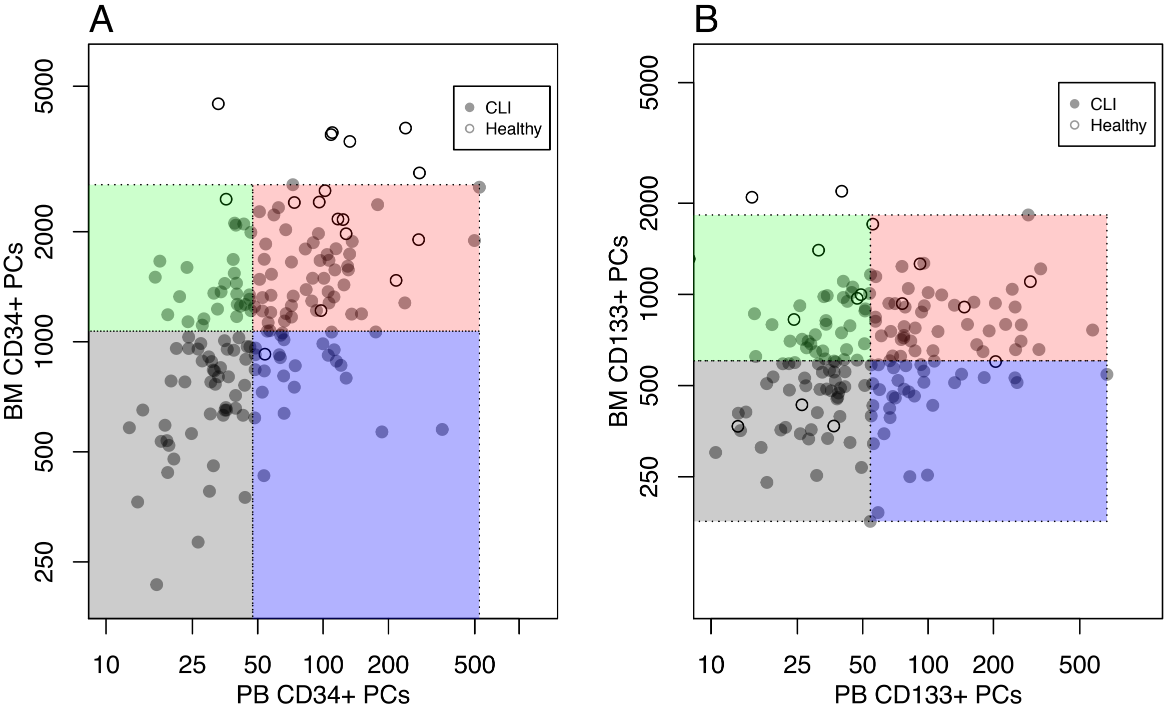

Figure 4: **Relative BM and PB PC numbers in CLI patients compared to Healthy Controls:** Figure shows BM PC number on y-axis and PB PC numbers on x-axis for 34+ PCs in panel **A** and CD133+ PCs in panel **B**. Closed circles denote CLI patients and open circles indicate healthy controls. The colored quandrants are based on the median values of the CLI patients for the respective cell populations as in main Figure 2. Healthy controls show relatively higher BM PC values ($p = 0.00002$ for CD34+ and $p = 0.003$ for CD133+), than PB PC numbers ($p = 0.003$ for CD34+ and $p = 0.61$ for CD133+). In addition, healthy control BM PC numbers exceed the range of CLI numbers in 6/17 cased for CD34+ PCs ($p = 0.018$) and 2/17 cases (ns) for CD133+ PCs; PB PC numbers showed a complete overlap in range.

Full Model

Risk Factor	PB CD34+ PCs		PB CD133+ PCs		BM CD34+ PCs		BM CD133+ PCs	
	HR	P-value	HR	P-value	HR	P-value	HR	P-value
**PCs**	0.71	0.07	0.62	0.012	0.49	0.03	0.65	0.16
**Age**	1.02	0.12	1.02	0.15	1.02	0.07	1.02	0.06
**Sex (Male)**	1.37	0.34	1.12	0.74	1.34	0.38	1.31	0.40
**GFR**	1.00	0.66	1.00	0.51	1.00	0.37	1.00	0.61
**History of CVA**	2.28	0.008	2.47	0.004	2.76	0.002	2.34	0.007
**Histor of MI**	1.41	0.22	1.48	0.16	1.33	0.31	1.35	0.28
**Ulcers**	1.92	0.04	1.99	0.03	1.95	0.038	1.98	0.032
**Triglycerides**	1.05	0.73	1.06	0.66	1.03	0.82	1.05	0.75
**Cholesterol**	1.01	0.92	1.01	0.92	1.05	0.72	1.01	0.94
**Likelihood Ratio test**		0.00004		0.00001		0.00003		0.00007

Most efficient models by backward factor Reduction

**PB CD34+ PCs**	Model factors: CD34+ PCs, Age, History of CVA, History of MI, Ulcers		**PB CD133+PCs**	Model factors: CD133+ PCs, History of CVA, History of MI, Ulcers	
Optimal Model:	AIC: 603.8		Optimal model	AIC 599.8	
Reduction			*Reduction*		
Model - CD34+PCs	AIC: 605.1	p = 0.07	Model - CD133+ PCs	AIC: 606.7	p = 0.003
Model - Age	AIC: 604	p = 0.14	Model - CVA	AIC: 608.9	p = 0.0009
Model - CVA	AIC: 609.5	p = 0.006	Model - MI	AIC: 601.1	p = 0.06
Model - MI	AIC: 604.6	p = 0.09	Model - Ulcers	AIC: 603.9	p = 0.014
Model - Ulcers	AIC: 607	p = 0.02			

**BM CD34+ PCs**	Model Factors: CD34+ BMPCs, Age, History of CVA, Ulcers		**BM CD133+ PC**	Model factors: CD133+ BMPCs, Age, History of CVA, Ulcers	
Optimal Model	AIC: 602.8		Optimal Model	AIC: 605.1	
Reduction			*Reduction*		
Model - CD34+ PCs	AIC: 606.0	p = 0.023	Model - CD133+ PCs	AIC: 606.0	p = 0.09
Model - Age	AIC: 604.7	p = 0.048	Model - Age	AIC: 607.2	p = 0.04
Model - CVA	AIC: 611.7	p = 0.001	Model - CVA	AIC: 612.1	p = 0.003
Model - Ulcers	AIC: 605.2	p = 0.036	Model - Ulcers	AIC: 608.0	p = 0.03

Table 1: **Adjusted Models:** Cox proportional hazards models corrected for Age, Sex, GFR, history of CVA, history of MI, ulcers, triglycerides and cholesterol are presented in the upper half of the table. In the lower half the results of automated backward exclusion of model factors based on AIC are presented. In each case the optimal model is given with associated AIC value, below the penalty of further exclusion of model factors are presented.
